# QuickStats

**Published:** 2015-01-30

**Authors:** FROM THE NATIONAL CENTER FOR HEALTH STATISTICS

**Figure:**
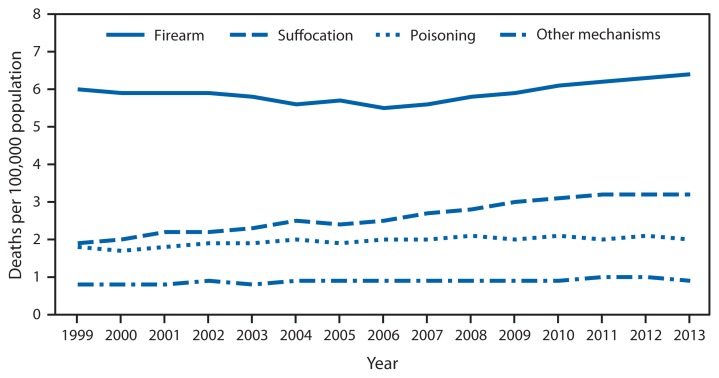
Suicide Rates,* by Mechanism of Injury^†^ — National Vital Statistics System, United States, 1999–2013 * Rates are age-adjusted using the 2000 U.S. standard population. ^†^ Suicide deaths were categorized by mechanism of injury using the following *International Classification of Diseases, 10th Revision* codes: firearm (X72–X74), suffocation (X70), poisoning (X60–X69), and other mechanisms (U03, X71, X75–X84, Y87.0).

From 1999 to 2013, the leading mechanism of injury for suicide for persons aged ≥5 years was firearm, followed by suffocation (including hanging) and poisoning (including drug overdose). During this period, the age-adjusted rate of suicide deaths by suffocation increased by nearly 70% from 1.9 per 100,000 in 1999 to 3.2 in 2013. In contrast, the suicide rates by firearm, poisoning, and other mechanisms remained relatively constant (6.0 per 100,000 in 1999 to 6.4 in 2013 for firearm; 1.9 per 100,000 in 1999 to 2.0 in 2013 for poisoning; and 0.8 per 100,000 in 1999 to 0.9 in 2013 for other mechanisms).

**Source:** National Vital Statistics System mortality data. Available at http://www.cdc.gov/nchs/deaths.htm.

**Reported by:** Yahtyng Sheu, PhD, ysheu@cdc.gov, 301-458-4354; Li-Hui Chen, PhD, Holly Hedegaard, MD.

